# Novel Staged Free-Fall Reactor for the (Catalytic) Pyrolysis of Lignocellulosic Biomass and Waste Plastics

**DOI:** 10.1021/acs.energyfuels.3c04733

**Published:** 2024-05-01

**Authors:** Songbo He, Jessi Osorio Velasco, Julian R. J. Strien, Zhenlei Zhang, Stefanie M. Bianchetti, Parniya Badr, Balaji Sridharan, Hendrik H. van de Bovenkamp, Robbie H. Venderbosch, Anton Bijl, Hero Jan Heeres

**Affiliations:** † Green Chemical Reaction Engineering, Engineering and Technology Institute Groningen, University of Groningen, Nijenborgh 4, Groningen 9747 AG, The Netherlands; ‡ Grupo de Termodinámica Aplicada y Energías Alternativas, Escuela de Procesos y Energía, Facultad de Minas, Universidad Nacional de Colombia, Carrera 80 No 65-223, Medellín 050034, Colombia; § CoRe Pro B.V., Osloweg 4-17, Groningen 9723 BL, The Netherlands; ∥ BTG Biomass Technology Group B.V., Josink Esweg 34, Enschede 7545 PN, The Netherlands; ⊥ Alucha Works B.V., Lange Linden 31, Cuijk 5433 NB, The Netherlands

## Abstract

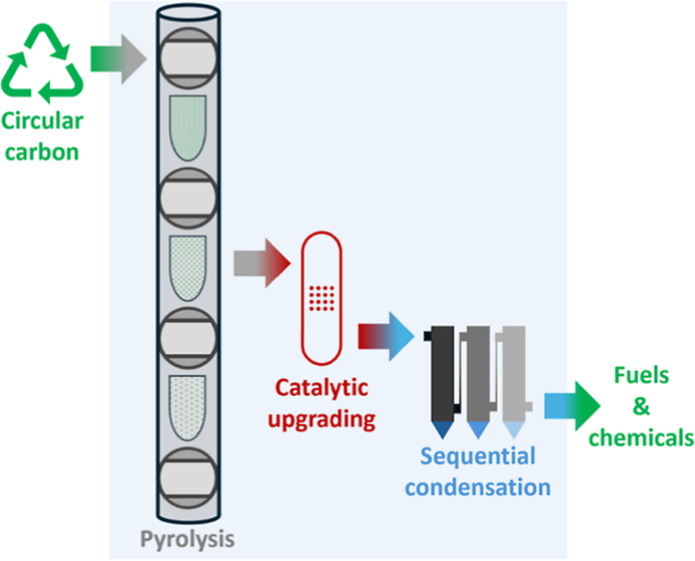

Pyrolysis of lignocellulosic biomass and waste plastics has been intensely studied in the last few decades to obtain renewable fuels and chemicals. Various pyrolysis devices have been developed for use in a laboratory setting, operated either in batch or continuously at scales ranging from milligrams per hour to tenths of g per hour. We report here the design and operation of a novel staged free-fall (catalytic) pyrolysis unit and demonstrate that the concept works very well for the (catalytic) pyrolysis of pinewood sawdust, paper sludge, and polypropylene as representative feeds. The unit consists of a vertical tube with a pretreatment section, a pyrolysis section, a solid residue collection section, a gas–liquid separation/collection section, and a catalytic reaction section to optionally perform ex situ catalytic upgrading of the pyrolysis vapor. The sample is placed in a tube, which is transported by gravity through various sections of the unit. It allows for rapid testing with semicontinuous feeding (e.g., 50 g h^–1^) and the opportunity to perform reactions under an (inert) gas (e.g., N_2_) at atmospheric as well as elevated pressure (e.g., 50 bar). Liquid yields for noncatalytic sawdust pyrolysis at optimized conditions (475 °C and atmospheric pressure) were 63 wt % on biomass intake. A lower yield of 51 wt % (on a biomass basis) was obtained for the noncatalytic pyrolysis of paper sludge, likely due to the presence of minerals (e.g., CaCO_3_) in the feed. The possibility of using the unit for ex situ catalytic pyrolysis (pyrolysis at 475 °C and catalytic upgrading at 550 °C) was also successfully demonstrated using paper sludge as the feed and H-ZSM-5 as the catalyst (21 wt % catalyst on biomass). This resulted in a biphasic liquid product with 25.6 wt % of an aqueous phase and 11 wt % of an oil phase. The yield of benzene, toluene, and xylenes was 1.9 wt % (on a biomass basis). Finally, the concept was also proven for a representative polyolefin (polypropylene), both noncatalytic as well as in situ catalytic pyrolysis using H-ZSM-5 as the catalyst at 500 °C. The liquid yield of thermal, noncatalytic plastic pyrolysis was as high as 77 wt % on plastic intake, while in situ catalytic pyrolysis gave a combined 7.8 wt % yield of benzene, toluene, and xylenes on plastic intake.

## Introduction

Fast pyrolysis of lignocellulosic biomass is an attractive technology to obtain biofuels and biobased chemicals. It involves the rapid heating of biomass in an oxygen-free atmosphere. During the process, cracking of the main components of lignocellulosic biomass (cellulose, hemicellulose, and lignin) into lighter compounds occurs, which, after condensation, gives a bioliquid known as pyrolysis oil or pyrolysis liquid.^1,2^ Biomass pyrolysis is a complex process in which many physical and chemical phenomena take place. Parameters such as feedstock (type and particle size), pyrolysis temperature, heating rate, and both solid and vapor phase residence time have major impacts on the yields and properties of the liquid product.^3^

Several biomass pyrolysis processes have been developed to date, using various reactor configurations such as fluidized bed reactors, free-fall reactors, rotating cone reactors, ablative pyrolysis reactors, wire-mesh systems, and auger-type reactors.^1,4−6^ Fluidized-bed reactors are widely used in lab-scale biomass pyrolysis systems with the characteristics of high heating rates, uniform temperature in the pyrolysis section, and high liquid yields (ca. 60–70 wt %).^1,7^ Application of these systems on a larger scale, however, is more complicated, and a large amount of fluidization gas is needed. This challenge is circumvented when using rotating cone technology, developed by BTG Biomass Technology Group B.V. and recently commercialized in The Netherlands, Finland, and Sweden.^8^ Another example is the fast pyrolysis reactor^9^ designed for biomasses containing large amounts of ash, e.g., paper sludge. Fast pyrolysis of paper sludge has been demonstrated by the authors on a lab-scale demonstrator (paper sludge feeding of ca. 11 kg h^–1^)^10^ and by Alucha Works B.V. on a pilot-scale unit (Mine 1, paper sludge feeding capacity of 100 kg h^–1^).

Laboratory equipment to perform research on biomass pyrolysis at technology readiness levels between 1 and 4 ranges from small-scale equipment using milligrams of biomass feed to continuous systems with biomass feeding rates up to 100 g h^–1^. Typical examples of the former are commercially available instruments such as the Pyroprobe 5200 (CDS Analytical)^11,12^ and tandem microreactor TMR Rx-3050TR (Frontier Laboratories).^13,14^ These systems operate in batchwise mode with respect to the biomass feed and do not allow for the collection of the products for off-line analyses and the determination of mass balance closures. Continuous lab scale systems have also been developed using, for instance, fluidized bed technology. These setups require the use of a dedicated transportation device (e.g., a screw conveyor) to feed the solid biomass to the pyrolysis reactor.^1,4,5,8^ However, this is challenging for laboratory-scale units due to the typically low feeding rates used (i.e., between 1 and 30 g h^–1^). Also, when lignin was used as the feed, blockage of the screws was reported due to coking. In addition, screw feeding does not (easily) allow for pyrolysis at elevated pressures (e.g., for hydropyrolysis purposes).

Recently, plastic pyrolysis has also received high attention to obtain fuels and chemicals.^15,16^ For polyolefins like polyethylene and polypropylene (PP), the main components in waste plastics, both thermal and catalytic options, have been explored. In the case of thermal pyrolysis, the prime objective is to obtain a liquid product with fuel or naphtha-type properties that can be used as such or cofed into an existing oil refinery.^17,18^ Catalytic options have also been developed, e.g., the catalytic pyrolysis of waste plastics to benzene, toluene, and xylenes (BTX).^19,20^

Based on the state of the art given above, there is an incentive to develop a multipurpose laboratory-scale pyrolysis system that allows for rapid testing without the need for screw feeding. Pyrolysis reactors without screw feeding have been reported in the literature. Examples include free-fall or drop-tube reactors. Here, the feed (biomass, plastics, etc.) is fed at the top of an empty heated tube and pyrolyzed while falling.^21−26^ For example, Ellens and Brown,^22^ reported a vertical stainless steel reactor consisting of a 1.8 m long pipe. The length is dictated by the time required to pyrolyze biomass particles with an average diameter of 400 μm in free-fall mode. Pyrolysis vapors are captured by condensers, including an electrostatic precipitator. However, a catalytic upgrading section is absent, and the pyrolysis vapors and the reactors cannot be operated at elevated pressures. Chen et al.,^27^ reported a lab-scale semicontinuous free-fall pyrolysis unit that is suited for the rapid testing of different feedstocks at a wide range of operating conditions (pressure and temperature), using different carrier gases (a.o. hydrogen and nitrogen), and optionally using a catalyst. The integrated reactor (7 m long) is composed of an upper drop-tube pyrolysis section and a lower moving-bed upgrading section. The unit allows for the introduction of a (biomass) source in the pyrolysis reactor without feeding complications.

**Figure 1:**
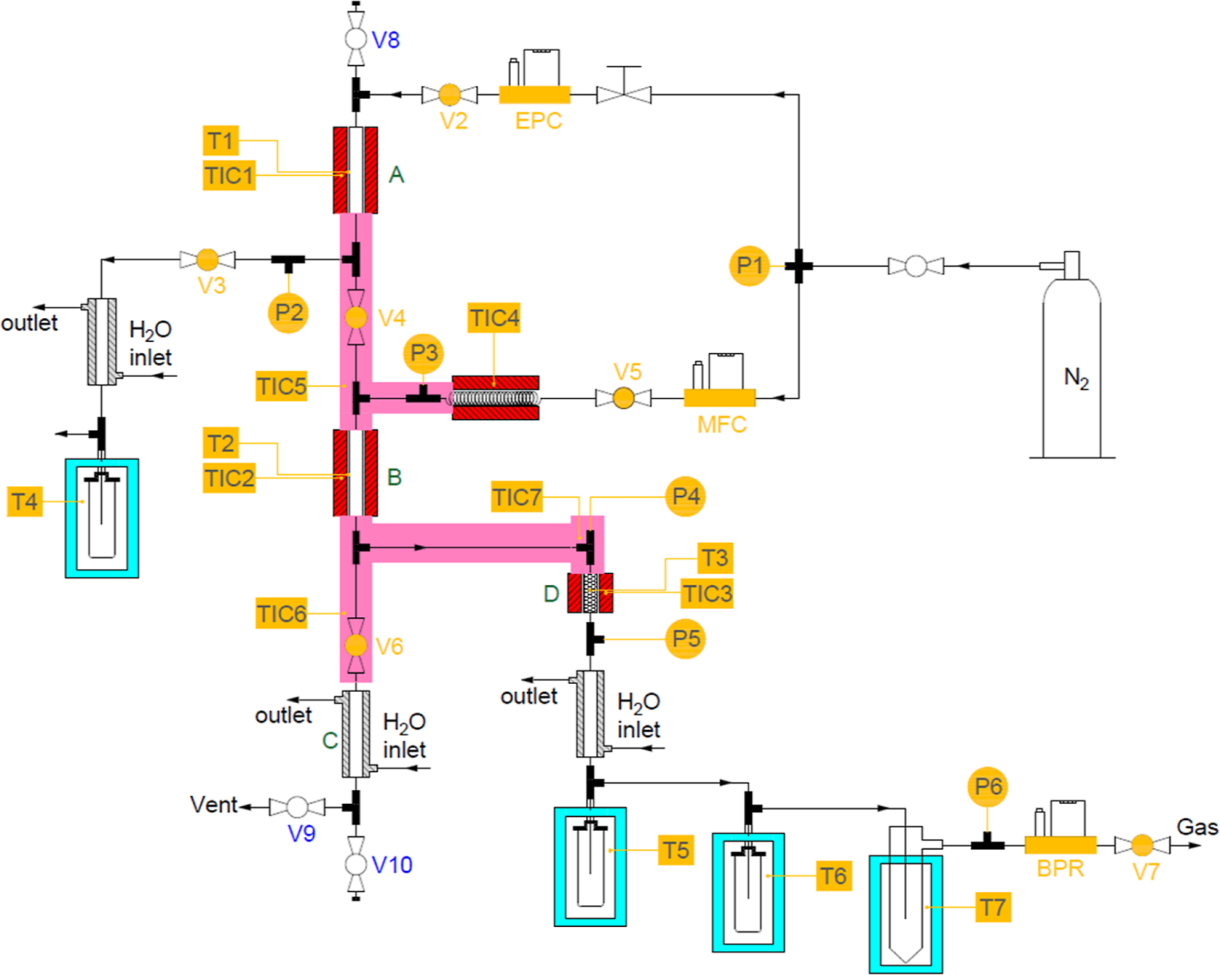
Schematic representation of the staged free-fall reactor.

In this work, we propose a novel pyrolysis unit that can be easily used in a laboratory environment. It involves a staged free-fall reactor,^22,27,28^ in which a vertical sample tube with one open and one closed end is used as the pyrolysis reactor. Herein, the feed within the tube falls through the various stages in the device by gravity (Figure 1). In this respect, it does not resemble a conventional free-fall or drop-tube reactor as the sample is present in a tube and the tube is passing through the various stages of the reactor. As such, the actual pyrolysis zone in the unit resembles a batch pyrolysis system, and, for example, the feed residence time in the pyrolysis zone is not dictated by the drop velocity of the (biomass) particles but can be set between minutes and hours, depending on the purpose of experimentation.

The novel staged free-fall pyrolysis concept allows for semicontinuous feeding of the selected feed to the pyrolysis reactor (which can be operated at pressures up to 80 bar) and both in situ and ex situ catalytic upgrading of the pyrolysis vapors. The pyrolysis of sawdust was performed as a benchmark at various pyrolysis temperatures, of which the optimized temperature was used for the pyrolysis of paper sludge. In addition, the ex situ catalytic pyrolysis of paper sludge using an H-ZSM-5 catalyst was investigated. Liquid pyrolysis products were analyzed by gas chromatography–mass spectrometry (GC–MS) to quantify the amounts of BTX. Finally, the use of the novel pyrolysis reactor for plastic pyrolysis was also investigated. Thermal pyrolysis of a representative waste plastic (PP) was performed, and the liquid products were analyzed in detail. In addition, the concept was also tested for in situ catalytic pyrolysis of PP using H-ZSM-5 as the catalyst, with the objective to obtain BTX.

## Experimental Section

### Materials

Pinewood sawdust (BEMAP-pine no. B03) was supplied by Bemap Houtmeel B.V. Paper sludge was supplied by Alucha Works B.V. Relevant properties are shown in Table 1. Tetrahydrofuran (THF, 99.85%) stabilized with butylated hydroxytoluene (BHT) (CAS: 109-99-9) was purchased from Biosolve. Di-*n*-butyl ether (DBE) was obtained from Sigma-Aldrich and was reagent grade (>99% purity). Toluene was supplied by Biosolve Chimie. *n*-Hexane 99% (CAS: 110-54-3) was purchased from Macron Fine Chemicals. Benzene 99.7% (CAS: 71-43-2) was purchased from VWR Chemicals. Toluene 99.85% (CAS:108-88-3) and pentane 96% (CAS: 109-66-0) were purchased from Biosolve. *m*-Xylene 99.9% (CAS: 108-38-3) was purchased from ABCR. *o*-Xylene 99.9% (CAS: 95-47-6) was purchased from ABCR. Carbon disulfide 99.9% (CAS: 75-15-0) and a C_7_–C_40_ saturated alkanes standard were purchased from Sigma-Aldrich (49452-U). Ammonia solution (25%) and hydrochloric acid solution (37–38%) were purchased from Boom Chemicals. Helium 5.0 and nitrogen 5.0 were purchased from Westfalen Gassen Nederland BV. 10% ammonia in helium was purchased from Linde Gas Benelux BV. PP (SABIC PP 571P) was obtained from Sabic. Relevant properties are given in Tables 1 and S1.

**Table 1:** Relevant Properties of the Sawdust, Paper Sludge, and Polypropylene Used in This Study

		sawdust	paper sludge	PP
moisture content (wt %)		7.9		
elemental composition (wt %)	C	47.1	27.5	85.2
	H	6.0	3.2	14.1
	N	0.1	0.3	
	S	0.01	0.4	
	others/O (by difference)	46.8	68.6	0.7
ash (wt %)		1.9	57	<0.1
particle size (mm)		0.05–0.5	0.05–0.5	0.05–0.5

Two H-ZSM-5 catalysts with different SiO_2_/Al_2_O_3_ molar ratios of 23 and 50, termed H-ZSM-5(23) and H-ZSM-5(50), were used and supplied by Zeolyst International. The received H-ZSM-5 powders were calcined before use at 550 °C for 5 h. For ex situ catalytic pyrolysis of paper sludge, H-ZSM-5(50) with a particle size of 0.6–1.2 mm was used to minimize the pressure drop in the fixed-bed reactor for catalytic upgrading of the pyrolysis vapor generated in the upstream pyrolysis reactor. For the in situ catalytic pyrolysis of polyolefins, H-ZSM-5(23) powder was used. A powdered catalyst was used to ensure homogeneous mixing of the catalyst and the polyolefin in the sample tube.

### Description of the Pyrolysis Unit

The pyrolysis unit was constructed by CoRe Pro B.V., The Netherlands.^29^ The core of the pyrolysis unit consists of a novel staged free-fall reactor made of 316 stainless steel tubing (1 × 0.083 in.). The reactor consists of three zones (i) a pretreatment section (e.g., to remove water and flush out air) (Figure 1A), (ii) a pyrolysis reactor (Figure 1B), and (iii) a solid residue collector (Figure 1C). The temperatures in the zones are independently controlled, and the zones are separated by two ball valves [Figure 1(V4,V6)]. The pyrolysis vapor optionally flows to a separate catalytic reactor (Figure 1D) made of 316 stainless steel tubing (0.5 × 0.104 in.), of which the temperature is also independently controlled. Afterward, the vapor product is cooled and then condensed in a three-stage condensation-separation system operated at different temperatures [Figure 1(T5–T7)] to collect the liquid products. The noncondensable gaseous product is collected in a gas bag for off-line analysis. N_2_ is typically used as the carrier gas, but it is possible to use other gases as well.

### Pine Wood and Paper Sludge Pyrolysis and Ex Situ Catalytic Vapor Upgrading

The preheater (A), pyrolysis reactor (B), and external catalytic reactor (D) are first heated and maintained at the desired temperatures under a flow of N_2_ (10–100 mL min^–1^). The temperatures for the three reactors are 100, 400–500, and 475 °C (550 °C for ex situ catalytic pyrolysis), respectively. A biomass sample [pine wood (7 g) or paper sludge (10 g)] in a stainless steel tube (top open and bottom closed) is introduced to the preheater (A) and thermally treated for 5 min (for sawdust pyrolysis) or 20 min (for paper sludge pyrolysis) to remove the volatiles (e.g., moisture). The volatiles are collected in a condenser [Figure 1(T4)]. The stainless steel tube containing the biomass is then transferred to the pyrolysis reactor (B) by quickly opening V4. Once the tube is inside the pyrolysis reactor, V4 is closed, and the pyrolysis reaction takes place for a fixed time of, e.g., typically 20 min for the pyrolysis of sawdust and paper sludge. The pyrolysis vapor flows to reactor D, which contains either no catalyst (for thermal pyrolysis experiments) or 4.5 g of catalyst (for ex situ catalytic pyrolysis experiments with paper sludge). The amount of catalyst is the maximum amount that can be loaded into the catalytic reactor section (D in Figure 1). For the ex situ catalytic experiments, the catalytic reactor with H-ZSM-5 was set at 550 °C. This temperature is based on the literature precedents and previous experience of our group on the (catalytic) pyrolysis of a wide range of biomass sources, such as glycerol,^30^ free fatty acids,^31^ and lignin.^32^ Afterward, the pyrolysis vapor is condensed in a set of three condensers maintained at 1, −20, and −50 °C, respectively [Figure 1(T5–T7)]. The liquid products from the condensers are collected and weighted, whereas the noncondensable gas is collected in a Tedlar bag for off-line gas analysis (GC-TCD). After pyrolysis, the tube containing solid residue is transported to a cooler (C) by gravity by switching on V6, and the tube is subsequently removed from the unit. After cooling to room temperature, the solid residue in the tube is weighed to calculate the mass balance. In a standard experiment, 3 to 5 “single shot” experiments are performed, and the total liquid product is collected in a single collection vessel. This way, sufficient liquid product is obtained to conduct various analyses, and experimental errors are minimized.

### Plastic Pyrolysis

A procedure similar to that given for biomass was also used for the pyrolysis of plastic (PP). Compared to biomass pyrolysis, the preheater is not used, as there is no moisture to be removed. The pyrolysis reactor and the catalytic upgrading reactor are set to 500 and 550 °C, respectively. Additionally, tracing between the interface of the pyrolysis reactor and the catalytic upgrading reactor is set to 300 °C to prevent condensation of the waxy product. The sample tube is filled with 10 g of PP and, in the case of catalytic pyrolysis, an additional 1 g of catalyst, and topped with a plug of steel wool. The total pyrolysis time was 1 h to ensure full conversion. In both cases, the catalytic upgrading reactor was not filled with catalyst.

The liquid collection is slightly different from that for biomass pyrolysis. The pyrolysis vapor is first cooled to 60 °C with the heat exchanger and is then condensed in the three condensers maintained at 20 (room temperature), −20, and −50 °C, respectively. Two single shot experiments are carried out per experiment, and the products are collected in different collection vessels.

### Determination of Product Yields

For each experiment, the liquid and solid products are weighed to determine the yields of pyrolysis oil and solids using eqs 1 and 2. For the (ex situ catalytic) pyrolysis of biomass, the solid residue collected from the pyrolysis reactor consists of only char. For the in situ catalytic pyrolysis of plastic, the solid residue consists of the used catalyst and char, of which the latter amount was determined using TGA. For the experiments using sawdust and plastics as feed, the weight of the gas product was not measured and is estimated from the liquid and char yields by eq 3, assuming a closed mass balance. The gas phase from the experiments using paper sludge as the feed (for experiments both without and with ex situ catalytic upgrading) was collected in a gas bag and weighed for mass balance closure calculations. The composition is determined by GC-TCD
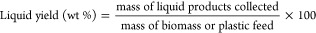
1
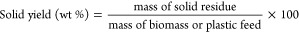
2
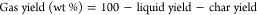
3

### Product Analyses

Elemental analyses (C, H, N, and S) were performed using a Euro Vector 3400 CHN-S analyzer. The oxygen content was determined by the difference. All analyses were carried out in duplicate, and the average value was reported.

GC-MS analysis of the liquid products of biomass pyrolysis is performed using a Hewlett-Packard 5973 MS attached to a Hewlett-Packard 6890 GC equipped with a Restek Rxi-5Sil MS column (30 m × 0.25 mm × 0.25 μm). The injection volume is 1 μL at an injector temperature of 280 °C. The oven temperature is kept at 45 °C for 2 min, raised to 280 °C with a ramping rate of 10 °C min^–1^, and then kept at 280 °C for 5 min. GC-FID analysis of plastic pyrolysis oils is performed on an Agilent 8860 GC equipped with a flame ionization detector (FID) and an Agilent HP-5 column of dimensions 30 m × 0.32 mm × 0.25 μm. The injection volume is 1 μL at an injector temperature of 280 °C. The oven temperature is set at 40 °C, raised to 325 °C with a ramping rate of 10 °C min^–1^, and then kept at 325 °C for 10 min. Quantification of BTX is done with a five-point calibration of benzene, toluene, *o*-xylene, and *m*-xylene in hexane. Prior to analysis, oil samples are dissolved in hexane at a dilution factor of 100.

The gaseous products collected using gas bags were analyzed and quantified on an HP 5890 GC-TCD equipped with a CP-PoraBOND Q column (50 m × 0.53 mm × 10 μm, supplied by Varian) and an HP-Molesieve column (30 m × 0.53 mm × 50 μm, supplied by Agilent). The composition was quantified using a standard gas containing known concentrations of H_2_, CO, CO_2_, C_1_–C_3_, and N_2_

The average molecular weight (*M*_n_ and *M*_w_) of the liquid products of biomass pyrolysis is determined by gel permeation chromatography (GPC) using an HP1100 instrument equipped with three MIXED-E columns (300 × 7.5 × 3 μm) in series using a GBC LC 1240 RI detector. THF is used as the eluent at a flow rate of 1 mL min^–1^, the pressure is set at 140 bar, the column temperature of 40 °C, and 20 μL injection volume with a 0.2 wt % sample concentration. Toluene is used as a flow marker. Polystyrene samples with different molecular weights are used as the calibration standards. Average molecular weight calculations are performed using the PSS WinGPC Unity software from the Polymer Standards Service.

Karl Fischer titrations using a Metrohm Titrino 758 model with Hydranal as the solvent are used for the determination of the water content of the samples.

Thermogravimetric analysis (TGA) was performed on a TGA 4000 instrument (PerkinElmer). The sample loaded into a ceramic crucible was heated to 800 °C with a heating rate of 10 °C min^–1^ under synthetic air (50 mL min^–1^).

## Results and Discussion

### Thermal Pyrolysis of Sawdust

Initial experiments on the thermal pyrolysis of sawdust were performed to determine the reproducibility of experimentation. For this purpose, three experiments were carried out with sawdust at a pyrolysis temperature of 450 °C. The liquid product yields were between 46.4 and 49.6 wt % with a standard deviation (SD) of 1.7%, whereas the solid product yields ranged between 32.7 and 33.6 wt % with a SD of 0.5%. These results indicate the good reproducibility of the pyrolysis experiments in the unit.

**Figure 2:**
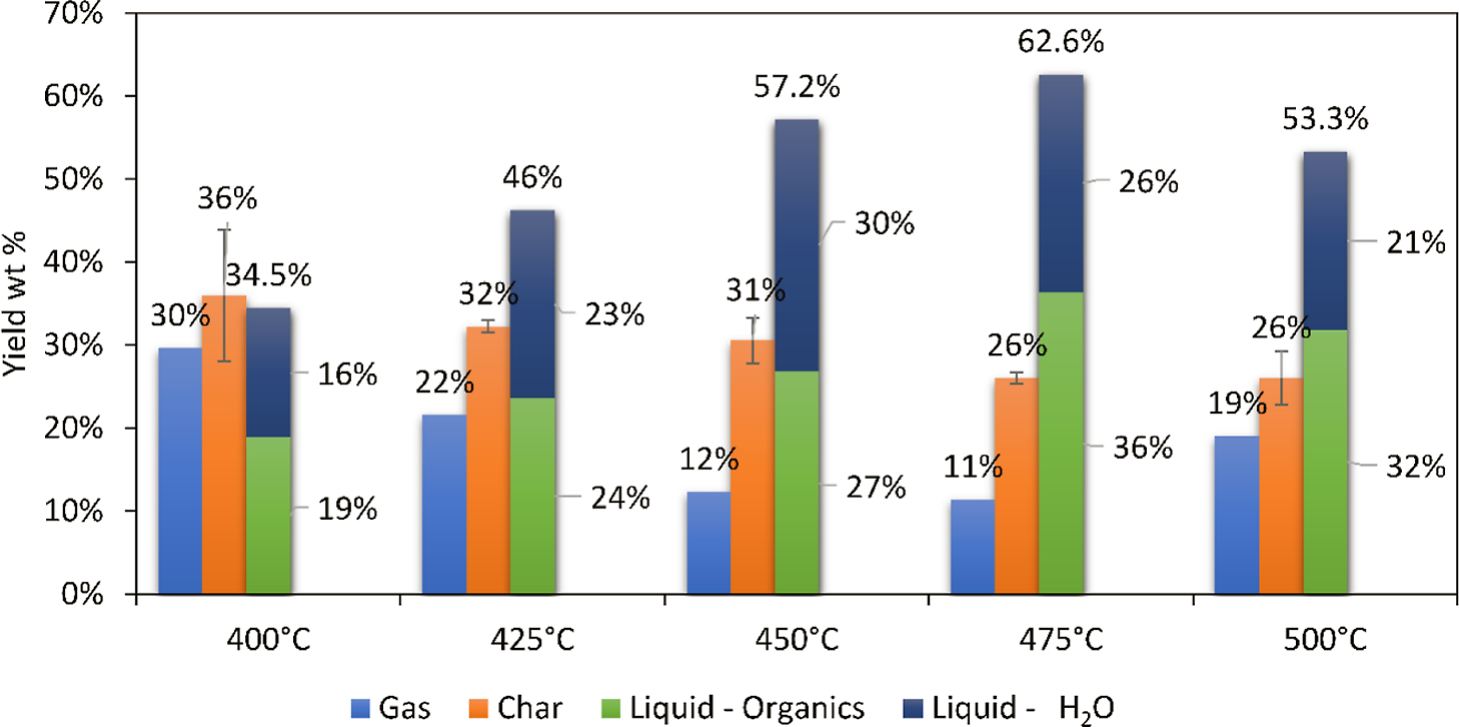
Product yields (wt % on biomass intake) of pyrolysis of sawdust at different pyrolysis temperatures.

Several pyrolysis experiments were performed with sawdust as the feed at different pyrolysis temperatures (i.e., 400, 425, 450, 475, and 500 °C), and the product yields are given in Figure 2. Relevant other process conditions are given in section—Pine Wood and Paper Sludge Pyrolysis and Ex Situ Catalytic Vapor Upgrading. Typically, a single-phase liquid product was obtained. The highest liquid yield was 62.6 wt % (ca. 36 wt % organics), obtained at 475 °C. Considering (i) the solid yield (26 wt %, Figure 2), (ii) a significant amount of water in the pyrolysis oil, and (iii) the relatively low heating rate of the sample in the pyrolysis reactor (ca. 1 °C/s, see Supporting Information), it appears that pyrolysis is occurring in the slow/intermediate regime considering the solid phase and fast with respect to the vapor phase (rapid removal of vapors from the pyrolysis section).^33,34^

A compilation of reported liquid yields for wood sawdust in various pyrolysis reactors is given in Table 2. Typically, the liquid yields are between 35% for slow pyrolysis and up to 70% for flash pyrolysis.

**Table 2:** Reported Pyrolysis Liquid Yields (wt %) for Wood Sawdust^35^

reactor	conditions	liquid yield (wt %)	ref.
spouted bed	455°C, 5–15 mm particle size	69	(36)
fixed bed	550°C, 50 g biomass intake (<1 mm)	46	(37)
fixed bed	500°C, 20 g biomass intake (0.2–1.8 mm particle size)	54	(38)
fluidized bed	500°C, 100 g h^–1^ biomass intake (<0.6 mm particle size)	62	(39)
fixed bed	500°C, 50 g biomass intake (<1 mm)	46	(40)
fixed bed	550°C, 15 g biomass intake	35	(41)

**Figure 3:**
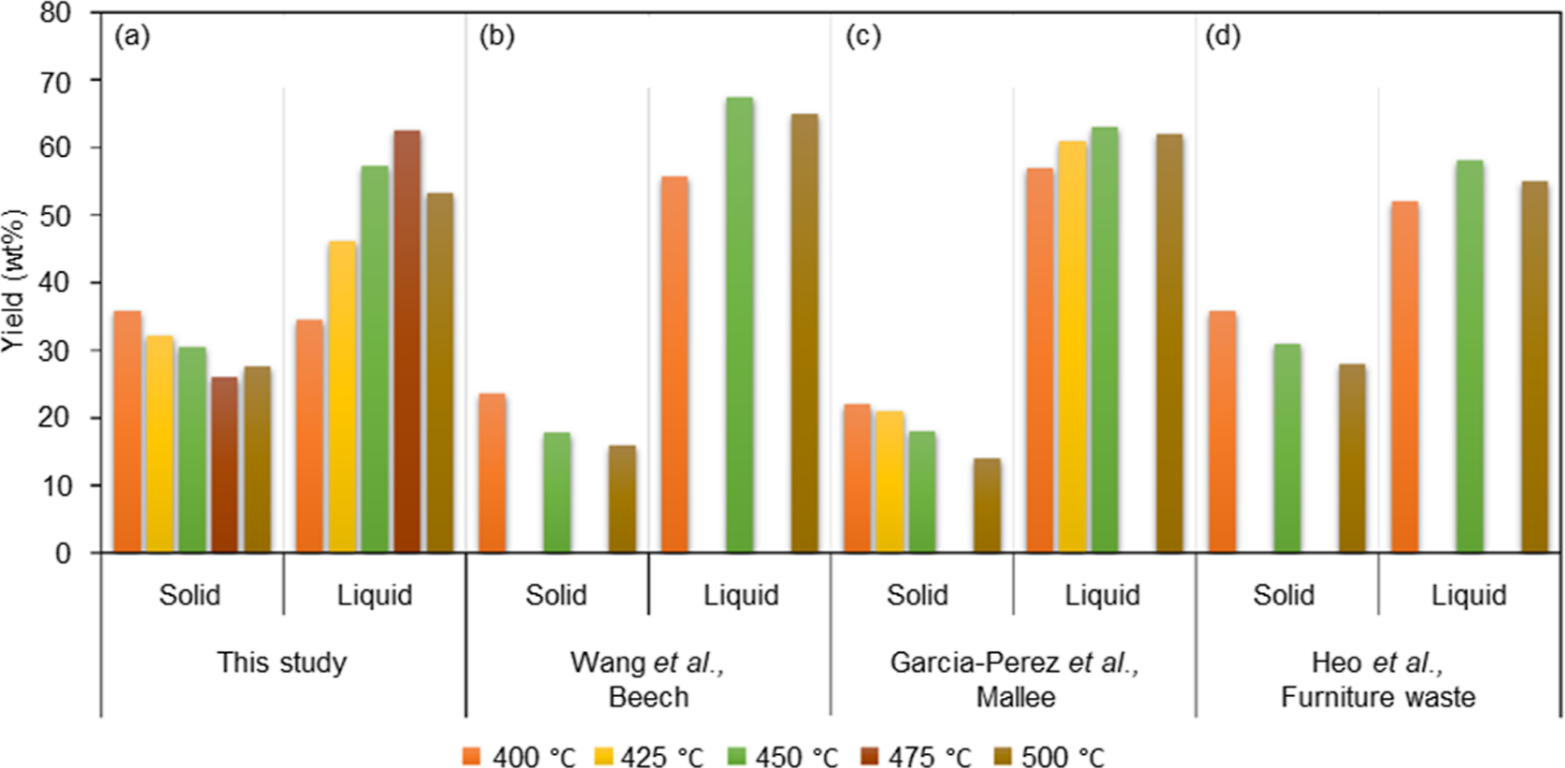
Comparison of the product yields obtained in the study (a) with representative examples reported in the literature (b,^43^ c,^44^ and d^34^) for woody biomass in fluidized bed reactors. (b) Reproduced with permission from ref (43). Copyright 2005, American Chemical Society. (c) Reproduced with permission from ref (44). Copyright 2008, American Chemical Society. (d) Reproduced with permission from ref (34). Copyright 2010, Elsevier.

The pyrolysis oil yields obtained in this study for wood sawdust are at the high end of the range, as reported in the literature, indicating that the staged free-fall reactor is a good alternative for biomass pyrolysis studies. Of interest is also a comparison of liquid yields with those obtained in fluidized bed reactors, which are characterized by a high heating rate of the feed and a low vapor residence time.^42^ A number of representative product yields obtained in such reactors using woody biomass as the feed are given in Figure 3.^34,43,44^ As such, the liquid yields are also in line with those obtained in typical fluidized bed reactors.

**Figure 4:**
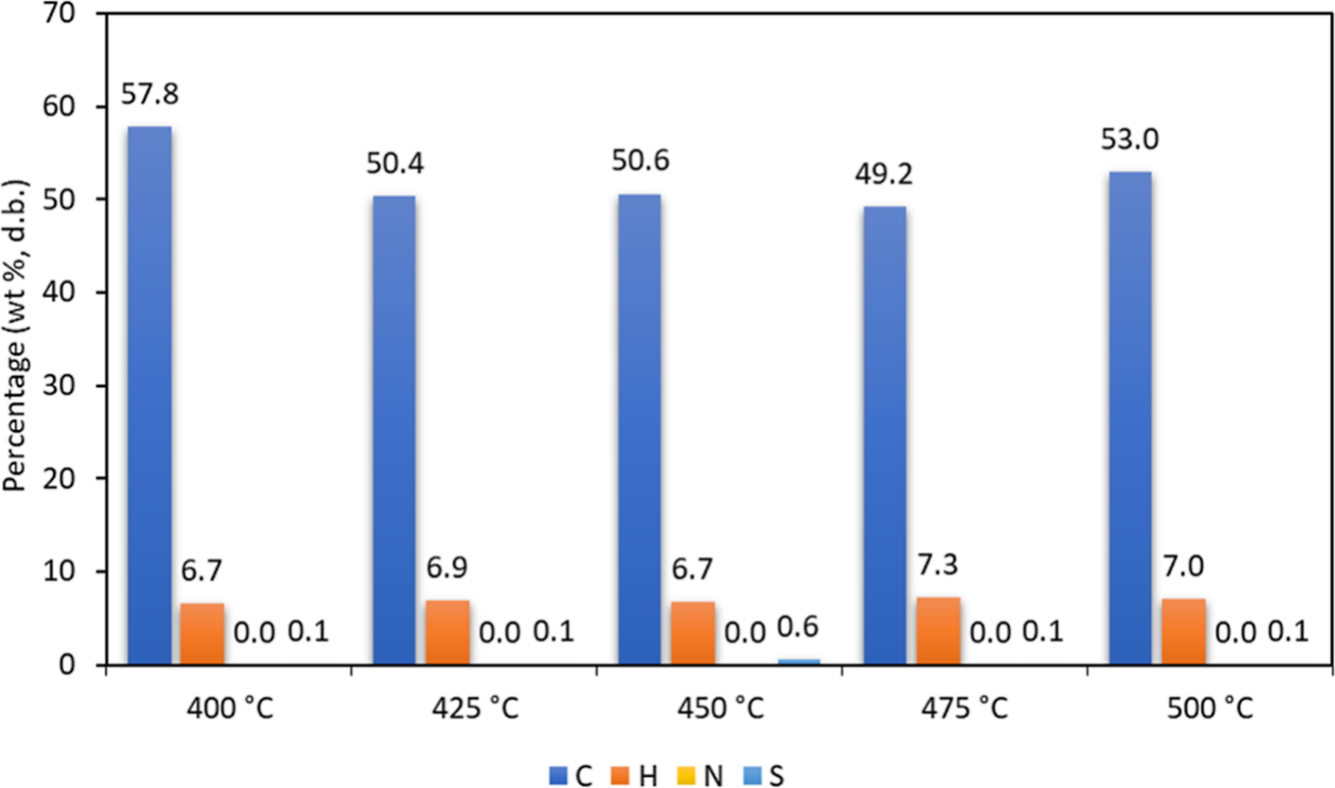
Elemental composition (dry basis) of the pyrolysis liquids obtained at different temperatures.

The pyrolysis liquids obtained at different pyrolysis temperatures using pinewood as the feed were analyzed in detail using elemental analyses, GC–MS, and GPC. The elemental composition of the pyrolysis liquid on a dry basis is shown in Figure 4. The carbon content (dry basis) is between 49 and 58%, which is in the range for typical pyrolysis liquids.^45^

The GC-FID chromatograms for the pyrolysis liquid obtained at 475 °C show the presence of typical wood-derived low molecular weight compounds such as 1-hydroxy-2-propanone, furfural, 2-hydroxy-2-cyclopenten-1-one (from the cellulose/hemicellulose fraction), and phenolics like phenol and substituted phenols from the lignin fraction,^46^ see Supporting Information (Table S2).

**Figure 5:**
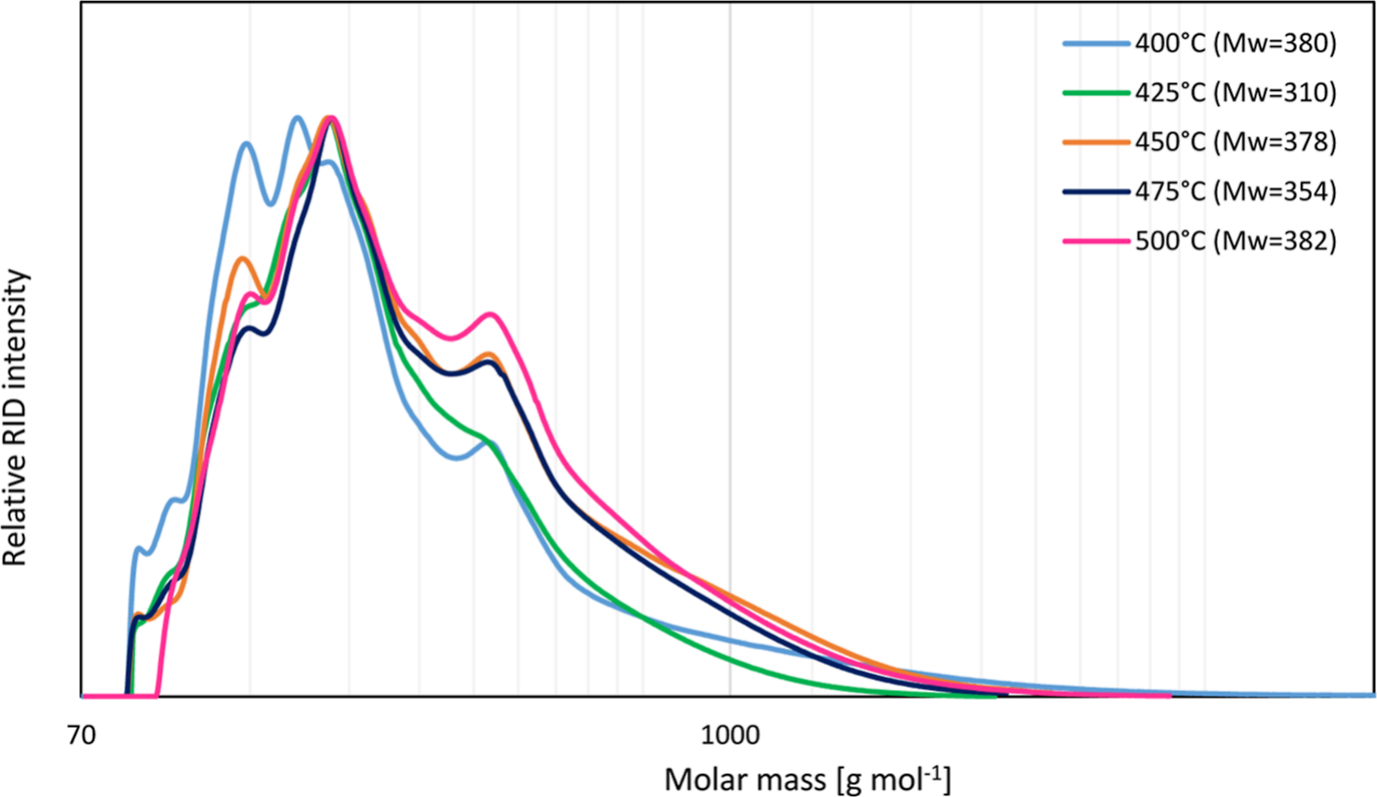
Temperature effect on the molecular weight distribution of the liquid phase for the pyrolysis of sawdust.

GPC (Figure 5) of the pyrolysis liquids provides insight into the non-GC detectable and shows a maximum in the low molecular weight region (80–300 g mol^–1^). However, significant tailing is observed, indicating the presence of higher molecular weight fragments in the oil. This tailing is particularly evident for pyrolysis liquids obtained at higher pyrolysis temperatures, which might be related to (i) repolymerization reactions in the vapor, which are favored at higher temperatures,^47^ and/or (ii) a higher rate of conversion of the lignin present in the biomass feed into oligomers. These oligomers are relatively heavy compounds, resulting in a liquid with a higher molecular weight tail.

### Pyrolysis and Ex Situ Catalytic Pyrolysis of Paper Sludge Using an H-ZSM-5 Catalyst

An interesting feature of the unit is the possibility to perform ex situ catalytic pyrolysis, for example, by using an H-ZSM-5 catalyst in the catalytic upgrading reactor (Figure 1D). For proof of concept, the ex situ catalytic pyrolysis of paper sludge was performed at 475 °C, and the pyrolysis vapor was passed over the H-ZSM-5(50) catalyst at 550 °C (9 wt % of catalyst on a biomass basis). The product yields are shown in Table 3, showing a good overall mass balance closure of 91.7 wt %. For comparison, the noncatalytic pyrolysis of paper sludge was also performed at the same conditions. This experiment also showed a good mass balance closure of 93.8% (Table 3). The pyrolysis liquid yield for thermal pyrolysis is about 51.1 wt % (on a biomass basis, Table 3), which is slightly lower than that for pinewood. This is very likely because of the catalytic effect of the minerals present in the paper sludge (e.g., CaCO_3_).^10^ For the catalytic pyrolysis of paper sludge, the pyrolysis liquid yield decreased to 37.0 wt %. This is attributed to the catalytic effect of H-ZSM-5, leading to a dramatically increased gas yield from 14.6 wt % for noncatalytic pyrolysis to 22.8 wt % for catalytic pyrolysis (both on a biomass basis, Table 3). As a result, a significantly higher BTX yield of 1.9 wt % was obtained for catalytic pyrolysis, compared to that for noncatalytic pyrolysis (0.16 wt %). Thus, we can conclude that the unit is also suitable for ex situ catalytic pyrolysis experimentation.

**Table 3:** Yields of the Solid, Liquid, and Gas, Mass Balances, and Yields of BTX for the Pyrolysis and Ex Situ Catalytic Pyrolysis of Paper Sludge

	pyrolysis	ex situ catalytic pyrolysis
solid (wt %)	65.6 (20.0)^a^	65.7 (20.0)^a^
liquid–oil (wt %)	4.8 (11.1)^a^	4.9 (11.4)^a^
liquid–aqueous (wt %)	17.2 (40.0)^a^	11.0 (25.6)^a^
coke on catalyst (wt %)		0.3 (0.7)^a^
gaseous products (wt %)	6.2 (14.6)^a^	9.8 (22.8)^a^
mass balance closure (%)	93.8	91.7
benzene (wt %)	0.06^a^	0.16^a^
toluene (wt %)	0.1^a^	0.98^a^
(*m*-)*p*-xylene (wt %)	0	0.65^a^
*o*-xylene (wt %)	0	0.11^a^
total BTX (wt %)	0.16^a^	1.9^a^

a Based on paper sludge input. Values in brackets are on a biomass basis.

### Pyrolysis and In Situ Catalytic Pyrolysis of Plastics

**Figure 6:**
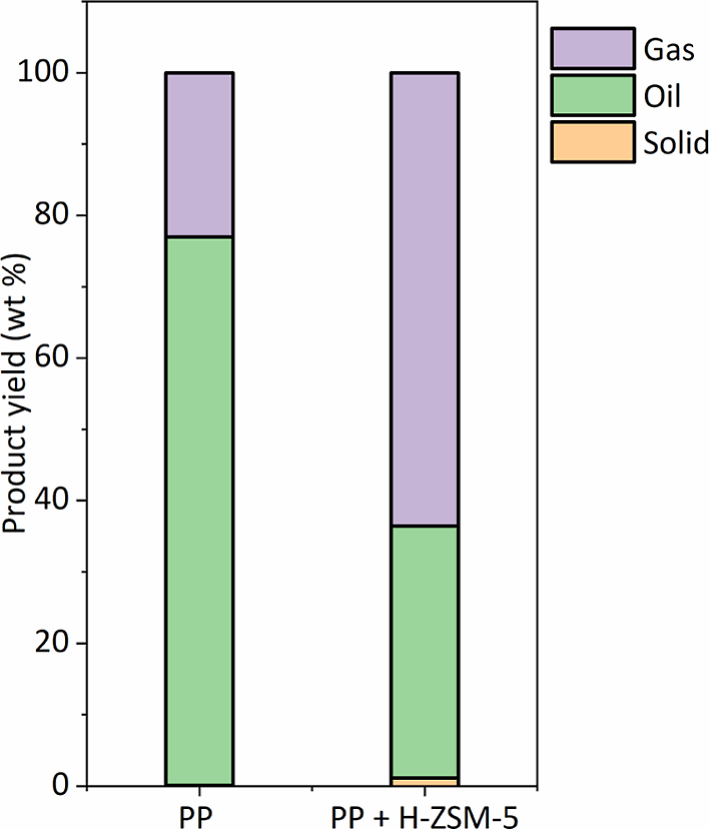
Mass balances for the thermal and in situ catalytic pyrolysis of PP (wt % on PP intake).

PP was pyrolyzed in both the absence (thermal pyrolysis) and presence of an in situ catalyst [H-ZSM-5(23)]. Relevant process conditions are given in section—Plastic Pyrolysis. Figure 6 shows the mass balance of the thermal and catalytic pyrolysis of PP. The highest liquid yield was obtained for thermal pyrolysis, viz., 76.9 wt % on intake, with a very small amount of solids (0.1%). The remaining 23.0 wt % is attributed to gas phase products, with the major components being propylene, ethylene, and methane. Catalytic pyrolysis yielded 32.3% of oil, 64.3% of gas products, and only minor amounts of char (<0.5%). Reproducibility was tested for thermal PP pyrolysis by performing 2 experiments at similar conditions (pyrolysis temperature of 500 °C). The liquid yield was found to be 76.0 ± 0.9%, showing good reproducibility.

For thermal pyrolysis of PP, reported liquid yields^48−51^ are up to 95 wt %, but these are known to be very dependent on temperature, heating rate, reactor type, and residence time.^48^ Liquid yields for the catalytic pyrolysis of PP over H-ZSM-5 zeolite catalysts are generally lower than 60%. Here, yields are a function of reaction conditions and are lower than those for thermal pyrolysis.^52−54^ Solid yields in this work are on the low end for the zeolite-catalyzed pyrolysis of polyolefins. Typically, solid yields are around 1–20% on intake, depending on the process conditions, catalyst loading, and reactor type.

**Figure 7:**
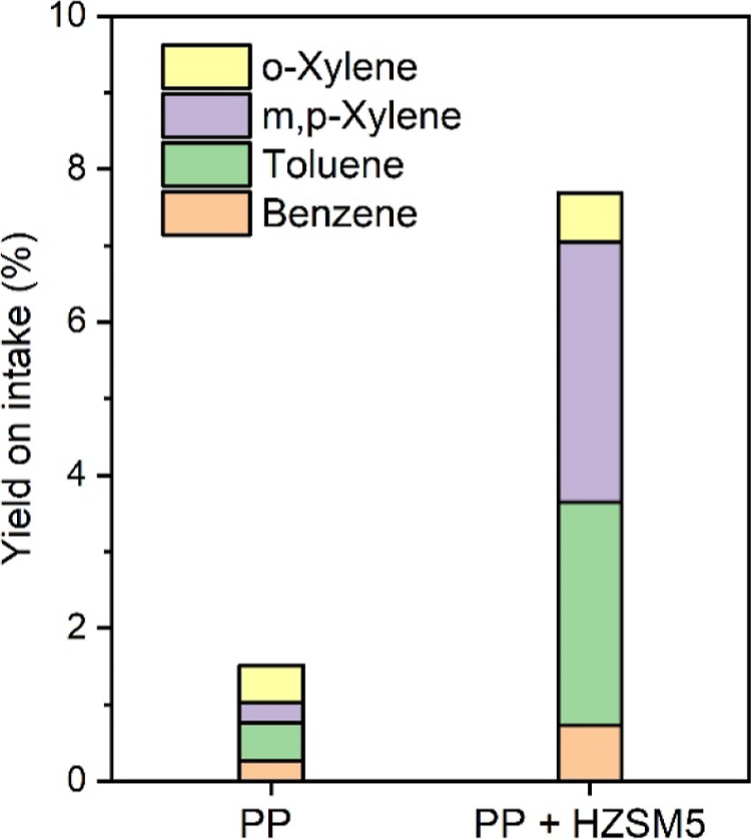
BTX yields (on PP intake) for thermal and in situ catalytic pyrolysis of PP using H-ZSM-5(23) (GC-FID with BTX calibration).

One-dimensional GC-FID was used to quantify the amounts of desired BTX in the oils, and the results are shown in Figure 7. For noncatalytic PP pyrolysis, small amounts of BTX are present (1.8 wt % on PP intake). In situ pyrolysis using H-ZSM-5 leads to a significant increase in BTX formation (7.8 wt % on PP intake). *M*,*p*-xylene and toluene are the main components, together with smaller amounts of benzene and *o*-xylene.

## Conclusions

We have designed, built, and successfully demonstrated the use of a novel (semi)continuous unit for the thermal, in situ, and ex situ catalytic pyrolysis of representative biomass and plastic feeds. It allows for rapid screening of feedstocks and catalysts with good mass balance closures and reproducibility, with the major advantage that screw feeding is not required. Liquid yields obtained for thermal pyrolysis of both pinewood sawdust (62.6 wt % at a pyrolysis temperature of 475 °C) and PP (76.9 wt % at 500 °C) are within the range reported for screw-type laboratory pyrolysis units. Catalytic pyrolysis experiments aiming for full deoxygenation to obtain BTX using both paper sludge (ex situ) and PP (in situ) revealed that the unit could also be used for this purpose. For the ex situ catalytic pyrolysis of PP with H-ZSM-5, a BTX yield of 7.8 wt % on PP was obtained. The full potential of the novel unit is currently being investigated by using other feedstocks such as technical lignins, which are notably difficult to pyrolyze in screw feed pyrolyzers, different atmospheres (e.g., hydropyrolysis using H_2_), elevated pressures (e.g., 50 bar), and molten salt-assisted pyrolysis, and the results will be reported in due course.

## Supporting Information Available

PP properties; sawdust pyrolysis liquid compounds; temperature profiles in the sample tube in the pyrolysis reactor including sample tube position in the pyrolysis reactor; sample tube bottom temperature versus time; and steady-state temperatures in the sample tube (PDF)
