# Supplementary material for: Novel Staged Free-Fall Reactor for the (Catalytic) Pyrolysis of Lignocellulosic Biomass and Waste Plastics
Source: Energy Fuels. 2024 May 1;38(10):8740–8. doi: 10.1021/acs.energyfuels.3c04733 (PMC11103701; doi:10.1021/acs.energyfuels.3c04733)
Supplement: Supplementary file 1 — ef3c04733_si_001.pdf [file ef3c04733_si_001.pdf]

Supporting information

A novel staged free-fall reactor for the (catalytic) pyrolysis of lignocellulosic biomass and waste plastics

Songbo He*[a,c], Jessi Osorio Velasco[a,b], Julian R.J. Strien[a], Zhenlei Zhang[a], Stefanie M. Bianchetti[a], Parniya Badr[a], Balaji Sridharan[a], Hendrik H. van de Bovenkamp[a], Robbie H. Venderbosch[d], Anton Bijl[e], Hero Jan Heeres[a,]*

[a]Green Chemical Reaction Engineering, Engineering and Technology Institute Groningen, University of Groningen, Nijenborgh 4, 9747 AG Groningen, The Netherlands

[b]Grupo de Termodinámica Aplicada y Energías Alternativas, Escuela de Procesos y Energía, Universidad Nacional de Colombia, Carr. 80 No. 65-223, Facultad de Minas, Medellín, Colombia

[c]CoRe Pro B.V., Osloweg 4-17, 9723 BL Groningen, The Netherlands

[d]BTG Biomass Technology Group B.V., Josink Esweg 34, 7545 PN Enschede, The Netherlands

[e]Alucha Works B.V., Lange Linden 31, 5433 NB Cuijk, The Netherlands

** Corresponding author's e-mail:* songbohe@gmail.com, h.j.heeres@rug.nl

Table S1. Relevant properties of the PP used.

Elemental CHNS		
	C%	85.2 ± 0.0
	H%	14.1 ± 0.1
	Other[1]	0.7 ± 0.1
TGA		
	Onset	421.7°C
	Maximum loss (inflection)	454.2°C
	98%	501.0°C
GPC		
	M_n (g mol^{-1})	60800
	M_w (g mol^{-1})	371700
	M_z (g mol^{-1})	1025000
	Polydispersity (M_w/M_n)	6.12
	Number average Degree of polymerization (M_n/M_0)	1444.9

[1] By difference

Table S2. List of compounds present in the pyrolysis liquid from the thermal pyrolysis of sawdust. (Reaction conditions: pyrolysis at 475°C for 20 min, pre-treatment at 100 °C for 5 min, N_2 flow of 18 ml min^{-1})

Compound
1- hydroxy-2-Propanone
Furfural
2-hydroxy-2-Cyclopenten-1-one
2(5H)-Furanone
Phenol
2-hydroxy-3-methyl-2-Cyclopenten-1-one
4-methyl-Phenol
2- methoxy-4-methyl-Phenol
1,2-Benzenediol
3- methyl-1,2-Benzenediol
4- methyl-1,2-Benzenediol
Eugenol
3-hydroxy-4-methoxy-Benzaldehyde
2-methoxy-4-(1-propenyl)-Phenol
2- methoxy-4-(1-propenyl)-Phenol
3- ethyl-2-hydroxy-2-Cyclopenten-1-one
Acetone
2-Cyclopenten-1-one
3-Furaldehyde
1,3-Cyclopentanedione
5-methyl- 2-Furancarboxaldehyde
3-methyl- 1,2-Cyclopentanedione
3-methyl-Phenol
2-methoxy-Phenol
2-Methoxy-5-methylphenol
1,4:3,6-Dianhydro-.alpha.-d-glucopyranose
4-ethyl-2-methoxy-Phenol
2-Methoxy-4-vinylphenol
2-methoxy-4-(1-propenyl)-Phenol
Vanillin
2,3-O-Acetonemannosan
1-(3-hydroxy-4-methoxyphenyl)-Ethanone
D-Allose
Acetic acid

Temperature profiles in the sample tube in the pyrolysis reactor

The temperature profile *versus* the time at the bottom of the sample tube when heating up in the pyrolysis section was measured using a thermocouple located within the tube. A schematic representation of the tube (resting on a spring) inside the pyrolysis reactor is shown in Figure S1. The tube and the spring (10 cm long) were dropped into the pyrolysis reactor, followed by the insertion of the thermocouple. The thermocouple was of K type (length of 1 meter and diameter of 3 mm) and the temperature was indicated by using a Wachendorff UR32742A PID controller. The temperature of the oven was maintained at 500 °C.

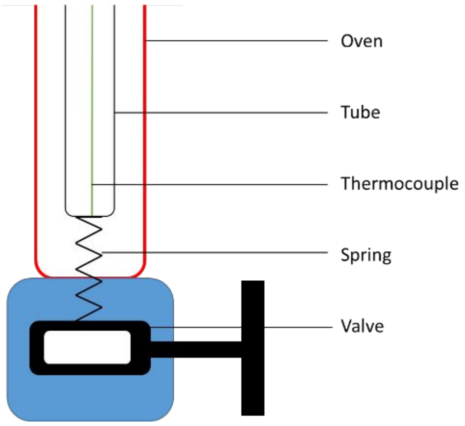

Figure S1. Schematic representation of the position of the sample tube in the pyrolysis reactor.

As can be seen in Figure S2, the temperature reached a steady temperature of 379°C after 17.5 minutes, however, 95% of this value (362°C) was reached in 8 minutes. Surprisingly, the temperature at this position in the tube is significantly lower than the oven temperature. This implies that the tube is not properly located in the oven and that the bottom part of the tube is outside the heated parts within the oven.

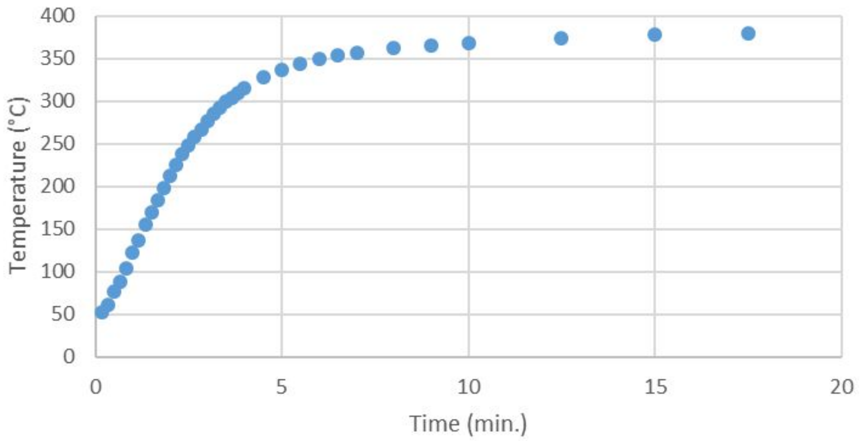

Figure S2. Temperature *versus* time at the bottom of the sample tube.

To study this, the thermocouple was lifted upwards with 10-cm increments to measure the temperatures at different locations in the tube (from the top to the bottom). The axial temperature profile is shown in Figure S3.

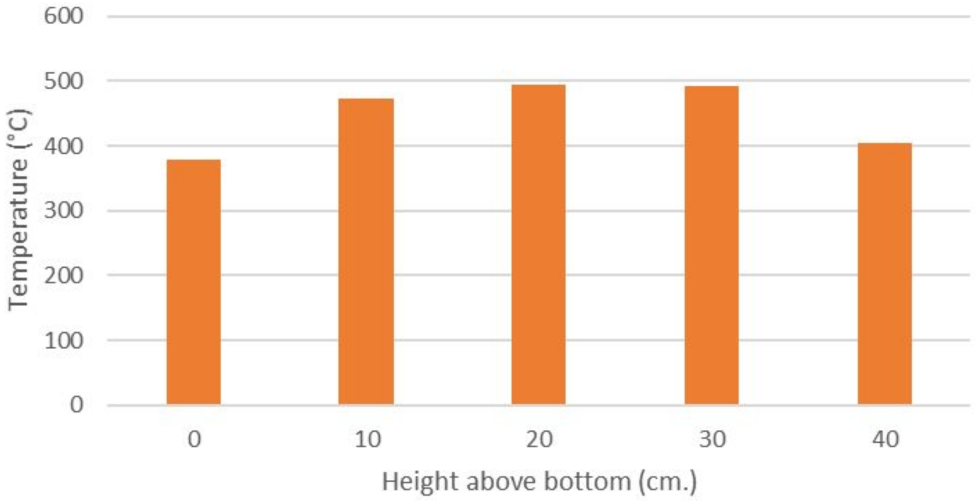

Figure S3. Steady-state temperature along the length of the sample tube.

According to this result, a long spring with a length of 20 cm was used in this study to locate the tube properly within the oven of the pyrolysis section. The temperature profile depicted in Figure S3 is based on an empty tube. During actual experiments, additional mass (i.e., sawdust) is present in the reactor, and thus the actual heating rate will be slightly slower. However, given the relatively low mass and specific heat capacity compared to that of the stainless-steel reactor tube, this additional heating time is expected to be very low.
